# Surgical Treatment of Cystic Adventitial Disease of the Popliteal Artery: Five Case Reports

**DOI:** 10.1155/2015/984681

**Published:** 2015-08-03

**Authors:** Kimihiro Igari, Toshifumi Kudo, Takahiro Toyofuku, Yoshinori Inoue

**Affiliations:** Division of Vascular and Endovascular Surgery, Department of Surgery, Tokyo Medical and Dental University, 1-5-45 Yushima, Bunkyo-ku, Tokyo 113-8519, Japan

## Abstract

Cystic adventitial disease (CAD) is a rare cause of intermittent claudication and nonatherosclerotic conditions in middle-aged men without cardiovascular risk factors. The etiology of CAD is unclear; however, the direct communication between a cyst and a joint is presumed to be a cause. We herein report a case series of CAD of the popliteal artery (CADPA), in which patients were treated with surgical resection and vascular reconstruction. Although less invasive treatment modalities, including percutaneous cyst aspiration and percutaneous transluminal angioplasty, have been the subject of recent reports, these treatments have had a higher recurrence rate. Therefore, all of the CAPDA cases in the present series were treated surgically, which lead to good outcomes.

## 1. Introduction

Cystic adventitial disease (CAD) is a rare nonatherosclerotic condition in which fluid accumulates subadventitially and compresses the lumen of the arteries and veins. In 80–90% of cases, CAD is located in the popliteal artery, where it may cause intermittent claudication and critical limb ischemia [1, 2]. The etiology of CAD has not been completely elucidated. It is hypothesized that a direct connection in the adventitia between the joint and the affected vessel grows into an abnormal cyst [3]. Due to this hypothesis, it is thought that CAD mainly affects the popliteal artery, which is located adjacent to the knee joint. CAD of the popliteal artery (CADPA) predominantly affects men of the ages of 40–50 years [4]. CADPA should differ from other peripheral arterial disease without the risk factors of cardiovascular diseases. In this report, we describe the results of our experience in the surgical treatment of CAD of the popliteal artery.

## 2. Case Presentation

### 2.1. Patients and Methods

A retrospective review was performed of all patients with a diagnosis of PFAA who underwent surgical treatment at Tokyo Medical and Dental University Hospital between January 2004 and December 2014. All subjects provided informed consent, and approval was obtained from our Institutional Review Board for a retrospective review of the patients' medical records and images. The diagnosis of CADPA was made by imaging methods including ultrasonography (US), computed tomography (CT), magnetic resonance imaging (MRI), and angiography. The medical records were abstracted to include basic demographic information, preoperative symptoms, surgical procedures, intraoperative findings, and long-term imaging findings. The characteristic features of the patients are listed in Table 1.

### 2.2. Case  1

**Figure 1:**
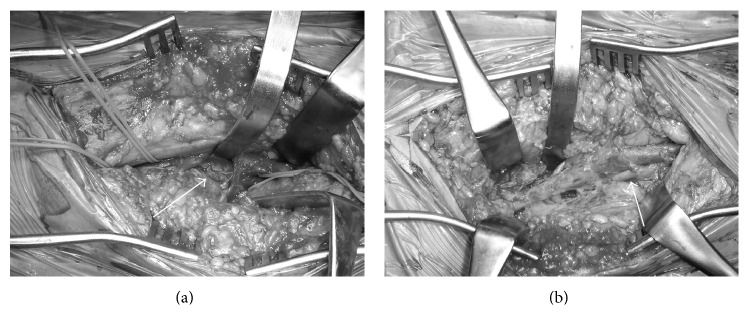
Intraoperative findings show (a) the controlled affected popliteal artery (white arrow) and (b) the resection being performed with an interposition graft (white arrow). The patient's head was to the right.

A 47-year-old male presented with a sudden-onset pain in his left leg and was admitted to another hospital. Angiography showed a 90% stenosis of the left popliteal artery, and he was transferred to our hospital. On physical examination, his left popliteal and pedal pulses were diminished, and his ankle brachial pressure index (ABI) on the left side was 0.5. US and MRI showed a severe stenosis of the left popliteal artery, which was compressed by a cystic mass. He was therefore diagnosed with CADPA. We decided to perform a surgical resection of the affected popliteal artery with vascular reconstruction. Under general anesthesia, his right great saphenous vein was harvested, and he was positioned prone for a posterior approach. The affected popliteal artery, including the cyst, was exposed and resected (Figure 1(a)), with revascularization using a harvested autologous vein graft (Figure 1(b)). The patient's postoperative course was uneventful. His postoperative ABI increased to 0.8. The histopathological findings showed fibrin and clots within the mucoid gel in the adventitia of the arterial wall, with an intact intima and media.

### 2.3. Case  2

**Figure 2:**
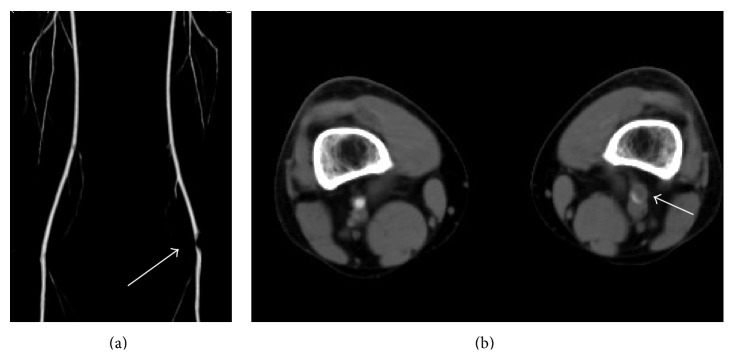
Computed tomography shows (a) the occlusion of the left popliteal artery (white arrow) and (b) a cystic mass compressing the popliteal artery (white arrow).

A 36-year-old male presented with an approximately one month history of intermittent claudication in his left calf with a symptom-free walk interval of 300 meters without rest pain. On physical examination, his left popliteal and pedal pulses were diminished, and his left ABI was 0.66. CT angiography showed an occlusion of the left popliteal artery (Figure 2(a)), compressed by a low density cystic mass (Figure 2(b)). Under general anesthesia, he was positioned prone to harvest the left great saphenous vein below the knee and expose the affected popliteal artery through a posterior approach. The occluded popliteal artery, with a compressing cystic lesion, was resected and the patient was interposed with great saphenous vein graft. The patient's postoperative course was uneventful without any evidence of lower limb ischemia. His postoperative ankle brachial pressure increased to 1.2.

### 2.4. Case  3

**Figure 3:**
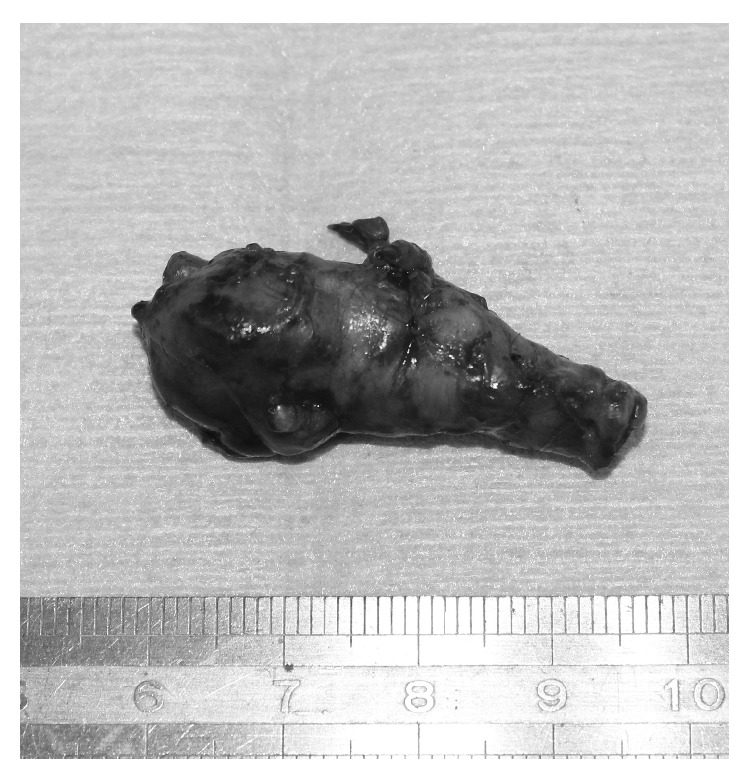
A resected specimen showing the popliteal artery with an adventitial cyst.

A 58-year-old male presented with one year history of intermittent claudication in his right calf with a symptom-free walk interval of 500 meters without rest pain. On physical examination, his right popliteal and pedal pulses were palpable, and his right ABI was within the normal range (1.1) at rest. However, after long-distance walking, his right popliteal pulse diminished. CT showed a stenosis of the right popliteal artery, compressed by a low density cystic mass. Under general anesthesia, he was positioned supine to harvest the ipsilateral great saphenous vein and expose the affected popliteal artery through a medial approach. The stenotic popliteal artery with a compressing cystic lesion was resected and the patient was interposed with a great saphenous vein graft. The patient's postoperative course was uneventful without any evidence of lower limb ischemia. His claudication after long-distance walking improved. CAD was confirmed by the histopathological findings, based on the presence of multiple mucinous foci of degeneration in the adventitia of arterial wall (Figure 3).

### 2.5. Case  4

**Figure 4:**
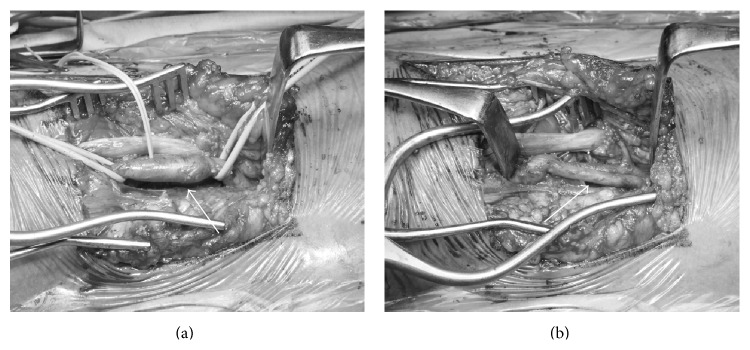
Intraoperative findings showing (a) the controlled left popliteal artery (white arrow) and (b) the performance of resection with graft interposition (white arrow). The patient's head was to the right.

A 63-year-old female presented with intermittent claudication in her left calf with a symptom-free walk interval of 100 meters without rest pain. On physical examination, her left popliteal and pedal pulses were diminished, and her left ABI was 0.87. CT showed a stenosis of the left popliteal artery, compressed by a low density cystic mass. Under general anesthesia, her right great saphenous vein was harvested, and she was positioned prone for a posterior approach. The affected popliteal artery, including the cyst, was exposed and resected (Figure 4(a)), with revascularization using a harvested autologous vein graft (Figure 4(b)). The patient's postoperative course was uneventful. Her postoperative ABI increased to 1.2.

### 2.6. Case  5

**Figure 5:**
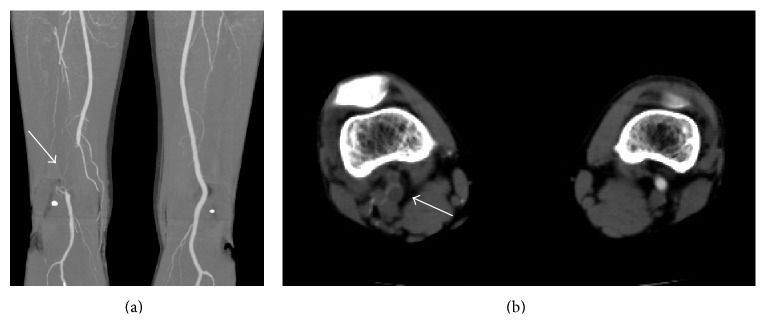
Computed tomography shows (a) the occlusion of the left popliteal artery (white arrow) and (b) a cystic mass compressing the popliteal artery (white arrow).

A 68-year-old male presented with intermittent claudication in his right calf with a symptom-free walk interval of 200 meters without rest pain. On physical examination, his right popliteal and pedal pulses were diminished, and his right ABI was 0.65. CT showed an occlusion of the right popliteal artery with a length of 6 cm (Figure 5(a)) and a cystic lesion which compressed the popliteal artery (Figure 5(b)). Under general anesthesia, he was positioned supine, and the affected popliteal artery was exposed through a medial approach. The occluded popliteal artery, including the cystic lesion, was resected and the patient was interposed with an 8 mm expanded polytetrafluoroethylene graft. The polytetrafluoroethylene graft was used because the patient's veins were small and unsuitable for the creation of an autologous graft. The patient's postoperative course was uneventful without any evidence of lower limb ischemia. His postoperative ABI increased to 1.11.

### 2.7. Surgical Procedures and Postoperative Results (Table 2)

A total of five CADPA patients were treated surgically. The mean operative time was 222 minutes (range: 200–262 minutes) and the mean amount of intraoperative blood loss was 180 mL (range: 82–432 mL); thus, none of the patients required a blood transfusion. Four of the five CADPA cases were interposed with a great saphenous vein graft. The other patient was interposed with an expanded polytetrafluoloethylene graft. A pathological examination of the resected popliteal arteries (including cysts) confirmed the diagnosis of CAD for each of the patients.

None of the patients exhibited lower limb ischemia after the surgical procedures and all were discharged successfully. During the long-term follow-up period (mean: 44 months, range: 10–124 months), no patients presented with signs of lower limb ischemia, and all of the interposed grafts remained patent.

## 3. Discussion

CAD remains a rare cause of lower limb ischemia, with a prevalence of 0.1% among the patients with intermittent claudication [4]. CAD mainly affects men, with a male to female ratio of 15 : 1. Patients with CAD first present clinical symptoms, including lower limb ischemia, between the ages of 10 and 70 years; the peak incidence is at 40 to 50 years [2]. In the present case series, most patients were male (80%), and the age at presentation ranged from 36 to 68 years. These findings are comparable with previous reports. Since CAD patients have no signs of atherosclerotic disease or cardiovascular risk factors; the diagnosis should be differentiated from popliteal artery entrapment syndrome, fibromuscular dysplasia, Buerger's disease, and popliteal artery aneurysm [5].

There are several hypotheses concerning the etiology of CAD, which is currently unclear. The proposed hypotheses include systemic disorders, repetitive trauma, an embryological origin, and the direct communication between a cyst and the adjacent joint (ganglion theory) [6]. Systemic disorders might be associated with generalized disorder, which leads to CAD; however, this hypothesis has failed to gain substantial support since the systemic manifestation of CAD has not been shown in the follow-up examinations of any patients [7]. Even though some authors have shown the traumatic events in patients with CAD, there remains a lack of young patients with CAD who have a history of repeated trauma. There was no history of recurrent trauma in the limbs of the patients in our case series, and it is difficult to decide the etiology of CAD as trauma. An embryological origin may be explained by the developmental theory, which states that mesenchymal mucin-secreting cell rests become incorporated within the adventitia of arteries during embryonic development [8]. However, this hypothesis is difficult to apply to the explanation of cyst recurrence after total cyst excision [9]. CAD occurs mainly in large arteries and veins which overlie a joint. Since Shute et al. first reported a direct communication between the knee joint and an adventitial cyst in 1973 [10], many authors have reported the same findings. This ganglion theory presumes that an adventitial cyst is the result of capsular synovial structures growing and tracking in the adventitia along the vascular branches. The theory is supported by the fact that the morphology of the cysts is very similar to that of ganglions in that they contain a high concentration of hyaluronic acid [11]. These direct communications have been found on preoperative imaging tests [12], and through intraoperative examinations [3]. This is now the most convincing and best-supported theory. Even though we did not find a pedicle around the cyst connected to the knee joint, the joint connections can be easily missed [13]; therefore, the communications may have been missed in the preoperative images and during intraoperative examination.

A typical clinical symptom of CADPA is the rapid progression of intermittent calf claudication, occasionally with sudden onset [14]. One of the 5 patients in our series presented with sudden-onset claudication; the other four patients presented with claudication that rapidly worsened. In some cases, normal pulses and normal ABI level have been associated with CADPA [15]. The ABI and pedal pulses in the affected side were normal at rest in the third case of our series; however, after exercise, the patient's ABI and pedal pulses diminished. Therefore, patients with a history of intermittent claudication and normal pedal pulses should be checked to differentiate CAD.

US, CT, MRI, and angiography are frequently used to diagnose CAD. MRI seems to be the most helpful of these modalities for detecting the relationship between cysts and vessels or the surrounding structures [16]. Furthermore, MRI can exclude other pathologies included in the differential diagnosis of the popliteal artery, such as atherosclerotic disease, aneurysm, popliteal artery entrapment syndrome, and soft-tissue tumors. MRI has also been demonstrated to be useful in detecting the connection between an adventitial cyst and the knee joint [17]. If MRI is employed more frequently in the diagnosis of CADPA, we might detect these direct connections. At present, however, the direct connection is thought to be too small to be revealed by any imaging techniques.

Several treatment options have been proposed for CADPA. These are divided into nonresectional and resectional techniques. Nonresectional techniques include open cyst evacuation with the removal of the cyst wall with or without patch angioplasty [18], percutaneous or open cyst aspiration, and percutaneous angioplasty [19]. Resection techniques consist of the resection of the affected artery and revascularization with direct anastomosis or graft interposition. While the aspiration technique is less invasive, the recurrence rate is high (approximately 40%) [1]. The endoluminal approach has an even higher recurrence rate (67%) [1]. These less invasive approaches are therefore considered to be inadequate for the treatment of CAD. Cyst evacuation has mostly been performed with nonresectional techniques, with an initial success rate of 94% [20]. However, these techniques are not suitable for the treatment of cases with total occlusion of the popliteal artery, which require vascular reconstruction. Therefore, the resectional techniques of cyst resection and vascular reconstruction are generally recommended as the treatment of choice for CADPA, especially in cases of total occlusion of the popliteal artery [2]. The recurrence rate of CAD treated by this technique is 1% [1], which is lowest rate of all of the treatment modalities. All of the cases in our series were therefore treated by the resection of the affected popliteal artery with autologous vein or prosthetic graft interposition. Furthermore, in resecting the affected popliteal artery, we are able to cut off the direct connection between the cyst and the knee joint, which is thought to create the adventitial cyst. This resectional technique is compatible from the point of view of CAD etiology.

In conclusion, we herein reported a case series of CADPA treated surgically with resection of the affected popliteal artery and vascular reconstruction in which treatment leads to good long-term outcomes. This resectional technique should be considered for the treatment of CAD, especially in cases in which there is an occlusion of affected vessels. Although CAD is a rare condition, it should be included in the differential diagnosis of young patients with intermittent claudication and no or poor comorbidities.

## Figures and Tables

**Table 1:** Patients characteristics.

Pt	Gender	Age	Laterality	Clinical symptoms	Diagnostic modality	Comorbidity
1	M	47	Lt	Rest pain, coldness	Angiography, US, MRI	DL, smoker
2	M	36	Lt	IC	CT	Smoker
3	M	58	Rt	IC	CT	HT
4	F	63	Lt	IC	US, CT	HT, smoker
5	M	68	Rt	IC	US, CT	None

^*∗*^Pt, patient; M, male; F, female; Rt, right; Lt, left; IC, intermittent claudication; US, ultrasonography; MRI, magnetic resonance imaging; CT, computed tomography; DL, dyslipidemia; HT, hypertension.

**Table 2:** Surgical procedures, intraoperative findings, and long-term follow-up results.

Pt	Surgical procedure	Conduit	Operative time (min)	Intraoperative blood loss (mL)	Follow-up (month)	Limb ischemia	Graft patency
1	Resection + revascularization	AVG	200	150	124	None	Patent
2	Resection + revascularization	AVG	213	432	52	None	Patent
3	Resection + revascularization	AVG	211	155	22	None	Patent
4	Resection + revascularization	AVG	262	84	16	None	Patent
5	Resection + revascularization	ePTFE	228	82	10	None	Patent

^*∗*^Pt, patient; AVG, autologous vein graft; ePTFE, expanded polytetrafluoroethylene.
